# Emergency Department Use by Youths Before and After Self-Inflicted Intentional Injury

**DOI:** 10.1001/jamanetworkopen.2024.27350

**Published:** 2024-08-15

**Authors:** Samaa Kemal, Rebecca E. Cash, Jennifer A. Hoffmann, Kenneth A. Michelson, Elizabeth R. Alpern, Margaret E. Samuels-Kalow

**Affiliations:** 1 Division of Emergency Medicine, Department of Pediatrics, Ann & Robert H. Lurie Children’s Hospital of Chicago, Northwestern University Feinberg School of Medicine, Chicago, Illinois; 2 Department of Emergency Medicine, Massachusetts General Hospital, Harvard Medical School, Boston

## Abstract

This cohort study describes the rate of emergency department (ED) encounters, reasons for these visits, and characteristics of the children and adolescents who seek this care.

## Introduction

Suicide is a leading pediatric preventable cause of death, and self-inflicted intentional injuries (hereafter, self-inflicted injuries) are associated with elevated suicide risk.^1^ Initiating prevention efforts in the emergency department (ED) is critical. Although most youth ED visits occur in general EDs,^2^ general EDs have fewer pediatric mental health resources.^3^ To situate ED-based interventions and understand disparities in outcomes, we examined ED use by youths before and after self-inflicted injury.

## Methods

We performed a retrospective cohort study of ED encounters by youths (aged 5-18 years) with self-inflicted injuries, as identified by *International Statistical Classification of Diseases, Tenth Revision, Clinical Modification* diagnosis codes, using Healthcare Cost and Utilization Project’s 2019 state ED and inpatient databases for Arkansas, Florida, Iowa, Maryland, Nebraska, New York, Vermont, and Wisconsin. Index injury encounter was considered the youth’s first ED encounter for a self-inflicted injury during the study period. We included states with high accuracy of the visit link variable, which permitted longitudinal follow-up. The Mass General Brigham Institutional Review Board deemed this study exempt from review and waived the informed consent requirement because deidentified data were used. We followed the STROBE reporting guideline.

Primary outcome was an ED encounter 90 days before or 90 days after the index injury. Primary exposure was ED type (pediatric or general) at the index injury encounter. Pediatric ED was defined by location in a freestanding children’s hospital or a hospital with a pediatric intensive care unit. All other EDs were considered general EDs. Additional variables included patient age, sex, race and ethnicity (Hispanic, non-Hispanic Black, non-Hispanic White, other), insurance payer, urbanicity, mechanism of injury, and ED disposition. Race and ethnicity were proxy for the implication of structural racism for health care use. The eMethods in Supplement 1 details the study methods.

We used generalized estimating equations, accounting for clustering of observations within hospitals, to identify associations between ED use and ED type. Analyses were performed using Stata SE, version 15.1 (StataCorp LLC).

## Results

We identified 15 593 ED encounters by youths (median [IQR] age, 15 [14-17] years; 11 484 females [73.6%], 4109 males [26.4%]; 12.4% Hispanic, 15.7% Black, 56.7% White, and 7.4% other race and ethnicity). Overall, 80.4% of patients lived in metropolitan areas, and 71.6% sought care at general EDs (Table 1).

**Table 1.** Characteristics of ED Index Injury Encounters for Self-Inflicted Injury by Youths

ED encounter characteristic	ED encounters for self-inflicted injury, No. (%) (N = 15 593)
Age group, y	
5-9	192 (1.2)
10-14	5426 (34.8)
15-18	9975 (64.0)
Sex	
Female	11 484 (73.6)
Male	4109 (26.4)
Race and ethnicity^a^	
Hispanic	1937 (12.4)
Non-Hispanic Black	2443 (15.7)
Non-Hispanic White	8840 (56.7)
Other^b^	1156 (7.4)
Missing data	1217 (7.8)
Urbanicity of youth residence	
Large metropolitan	6640 (42.6)
Small metropolitan	5894 (37.8)
Micropolitan	1708 (11.0)
Rural	1329 (8.5)
Insurance status	
Private	6240 (40)
Public	8172 (52)
Other^c^	1181 (8)
Mechanism of injury	
Cut or pierce	4695 (30.1)
Poisoning, drug	8805 (56.5)
Poisoning, nondrug	467 (3.0)
Other specified^d^	1966 (12.6)
Unspecified	1628 (10.4)
ED type at index injury encounter^e^	
Pediatric ED	4431 (28.4)
General ED	11 162 (71.6)
ED disposition for index injury	
Admission	4030 (25.8)
Discharge (to home or non–acute care)	8298 (53.2)
In-hospital mortality	38 (0.2)
Transfer to acute care	1290 (8.3)
Transfer from general to pediatric ED^e^	481 (3.1)
Transfer to psychiatric hospital	3217 (20.6)

Abbreviation: ED, emergency department.

^a^ Mutually exclusive race and ethnicity categories were identified from the Healthcare Cost and Utilization Project race variable.

^b^ Other race and ethnicity include Asian or Pacific Islander, Native American, and other.

^c^ Other includes self-pay, no charge, other, and missing data.

^d^ Other specified was defined as occurring in less than 2% of the sample.

^e^ Pediatric ED was defined as an ED located in a freestanding children’s hospital or a hospital with a pediatric intensive care unit. All other EDs were categorized as general.

Within 90 days before an index injury, 24.2% of youths had an ED encounter, of which 71.8% occurred in general EDs and 38.2% were for mental or behavioral health. Adjusted odds of having an ED encounter within 90 days before an index visit was higher among youths with public insurance (adjusted odds ratio [AOR], 1.77; 95% CI, 1.60-1.96) and youths living in rural areas (AOR, 1.31; 95% CI, 1.10-1.57) (Table 2).

**Table 2.** Adjusted Odds of ED Encounter 90 Days Before or 90 Days After Encounter for Self-Inflicted Injury

ED encounter characteristic	ED encounter for self-inflicted injury, AOR (95% CI)	
	90 d Before injury (n = 11 070)	90 d After injury (n = 11 701)
Age group, y		
5-9	1 [Reference]	1 [Reference]
10-14	0.80 (0.55-1.16)	1.09 (0.77-1.54)
15-18	0.91 (0.62-1.33)	1.05 (0.75-1.47)
Sex		
Male	1 [Reference]	1 [Reference]
Female	1.01 (0.91-1.12)	1.01 (0.91-1.11)
Race and ethnicity^a^		
Hispanic	0.88 (0.75-1.02)	0.81 (0.69-0.94)
Non-Hispanic Black	1.09 (0.95-1.25)	0.97 (0.85-1.11)
Non-Hispanic White	1 [Reference]	1 [Reference]
Other^b^	0.85 (0.70-1.04)	0.85 (0.71-1.02)
Missing data	0.79 (0.61-1.02)	1.09 (0.89-1.33)
Urbanicity of youth residence		
Large metropolitan	1 [Reference]	1 [Reference]
Small metropolitan	1.12 (0.99-1.27)	1.11 (0.99-1.25)
Micropolitan	1.06 (0.90-1.24)	1.29 (1.10-1.50)
Rural	1.31 (1.10-1.57)	1.21 (1.02-1.44)
Insurance status		
Private	1 [Reference]	1 [Reference]
Public	1.77 (1.60-1.96)	1.72 (1.57-1.90)
Other^c^	1.15 (0.96-1.38)	1.13 (0.93-1.36)
ED type at index injury encounter^d^		
General ED	1 [Reference]	1 [Reference]
Pediatric ED	1.05 (0.92-1.20)	1.06 (0.93-1.20)

Abbreviations: AOR, adjusted odds ratio; ED, emergency department.

^a^ Mutually exclusive race and ethnicity categories were identified from the Healthcare Cost and Utilization Project race variable.

^b^ Other race and ethnicity include Asian or Pacific Islander, Native American, and other.

^c^ Other includes self-pay, no charge, other, and missing data.

^d^ Pediatric ED was defined as an ED located in a freestanding children’s hospital or a hospital with a pediatric intensive care unit. All other EDs were categorized as general.

Within 90 days after an index injury, 26.7% of youths had an ED encounter, of which, 69.2% occurred in general EDs and 47.9% were for mental or behavioral health. Adjusted odds of having an ED encounter within 90 days after an index visit was higher among youths with public insurance (AOR, 1.72; 95% CI, 1.57-1.90), youths living in rural areas (AOR, 1.21; 95% CI, 1.02-1.44), and youths living in micropolitan areas (AOR, 1.29; 95% CI, 1.10-1.50) (Table 2).

## Discussion

In this retrospective cohort study, we found high rates of ED use by youths before and after self-inflicted injury. This finding suggests an opportunity for ED-based interventions, such as suicide risk screening, safety planning, and linkage to services.^4^

We also found that most youths, especially those in rural areas and with public insurance, received care exclusively at general EDs. These youths are disproportionately affected by pediatric mental health professional shortages^5^ and decreased access to specialized pediatric care.^6^

Study limitations included the potential for misclassification bias when using diagnosis codes, residual confounding on variables unavailable in the dataset, and poor generalizability, given that findings were limited to 8 states. Further work to implement preventive interventions in both pediatric and general EDs is warranted to serve high-risk populations equitably and effectively.

## Notes

Supplement 1. **eMethods.** Expanded Description of Inclusion Criteria, Exposures and Analytic Approach
eReferences.

Supplement 2.
Data Sharing Statement
