# Supplementary material for: Emergency Department Use by Youths Before and After Self-Inflicted Intentional Injury
Source: JAMA Netw Open. 2024 Aug 15;7(8):e2427350. doi: 10.1001/jamanetworkopen.2024.27350 (PMC11327883; doi:10.1001/jamanetworkopen.2024.27350)
Supplement: Supplement 1. — eMethods. Expanded Description of Inclusion Criteria, Exposures and Analytic Approach eReferences. [file jamanetwopen-e2427350-s001.pdf]

Supplemental Online Content

Kemal S, Cash RE, Hoffmann JA, Michelson KA, Alpern ER, Samuels-Kalow ME. Emergency department use by youths before and after self-inflicted intentional injury. *JAMA Netw Open*. 2024;7(8):e2427350. doi:10.1001/jamanetworkopen.2024.27350

eMethods. Expanded Description of Inclusion Criteria, Exposures and Analytic Approach.

eReferences.

This supplemental material has been provided by the authors to give readers additional information about their work.

© 2024 Kemal S et al. *JAMA Network Open*

eMethods. Expanded Description of Inclusion Criteria, Exposures and Analytic Approach.

Inclusion Criteria. We included all Emergency Department (ED) encounters for youth (5-18 years old) with an *International Classification of Diseases, Tenth Revision, Clinical Modification* (ICD-10-CM) code for self-inflicted intentional injury, defined by any “Suicide or Self-Injury” code in the Child and Adolescent Mental Health Disorders Classification System (CAMHD-CS)[1,2] except R45.851 (suicidal ideations). Two overlapping cohort subsets were defined based on the time of index injury to permit uniform lookback and follow-up durations. Subset 1 included patients with the opportunity to have an ED encounter in any of the 90 days before their index injury (i.e., patients with an index injury from April-December); Subset 2 was defined as patients with the opportunity to have an ED encounter in any of the 90 days after their index injury (i.e., index injury from January-September).

Variables. We defined the type of ED based on pediatric resources available at the hospital and categorized EDs as being pediatric or general. Pediatric EDs included EDs located within a) freestanding children’s hospitals which had ≥70% visits by children and/or restricted admissions to children in American Hospital Association (AHA) data and b) hospitals that did not meet the qualification for a freestanding children’s hospital but had a pediatric intensive care unit (PICU). PICU admissions were determined by critical care charges during an admission for patients aged 1-15 years. The lower age cutoff was to avoid PICU determination based on NICU admissions. Presence of a PICU required having >25 critical care admissions. Visit volume and admissions were determined directly from the State Emergency Department Databases and State Inpatient Databases (SEDD/SID). For states where required data regarding critical care charges were not available (e.g., Vermont), we used AHA data to determine PICU capabilities. Only one hospital was included using this strategy. All EDs that did not meet the qualifications for a pediatric ED were considered a general ED.

We also acquired the following data for each encounter. Patient age was categorized as 0-4 years, 5-9 years, 10-14 years, and 15-18 years. Sex was categorized as male or female. Mutually exclusive race and ethnicity categories were determined using the Healthcare Cost and Utilization Project (HCUP) “RACE” variable,[3] a uniform coding for race and ethnicity, as follows: Hispanic, non-Hispanic Black, non-Hispanic White, and other. “Other” encompassed categories representing <5% of the study sample: “Asian or Pacific Islander”, “Native American”, and “other.” Recognizing that race and ethnicity are social and not biological constructs, we included this variable as a proxy for the influence of structural racism on healthcare utilization, which may contribute to healthcare disparities.[4] Payer was categorized as private, public, or other. Urbanicity of patient residence was determined using the HCUP urban-rural designation, adapted from 2013 Urban Influence Codes,[5] stratified as: large metropolitan area (≥1 million residents), small metropolitan (<1 million residents), micropolitan, and not metropolitan or micropolitan (i.e., rural). Mechanisms of injury were classified using the Centers for Disease Control and Prevention External Cause of Injury Matrix.[6] Mechanisms occurring in <2% of the study sample were classified as “Other Specified.” We described disposition from the ED including whether a patient was admitted, discharged, transferred to another ED (including whether transfer was from a non-pediatric to pediatric ED), transferred to a psychiatric hospital (or psychiatric distinct unit of a hospital), or if the patient had in-hospital mortality (including mortality in ED or inpatient). We determined proportion of visits for “mental or behavioral health complaints” using the HCUP Clinical Classification Software Refined coding system.[7]

Statistical Analysis. We calculated descriptive statistics to summarize youth and encounter characteristics at time of the index injury and the characteristics of encounters before (Subset 1) and after (Subset 2) the index injury. We used generalized estimating equations (GEE) with a binomial distribution, logit link, working independence correlation structure, and robust standard errors, to examine associations of patient characteristics (age, sex, race and ethnicity, urbanicity, payer) and ED type at index injury with ED utilization outcomes, adjusting for clustering of observations within hospitals. We assessed two primary outcomes in separate models: (1) any ED visit in the 90 days before index injury (Subset 1); (2) any ED visit in the 90 days after the index injury (Subset 2). Youth who died at the index visit were excluded from the analysis of ED visits after injury. There were minimal missing data in this dataset. For race and ethnicity and insurance status, missing data were coded as a category. Otherwise, available case analysis was used to handle missing data.

© 2024 Kemal S et al. *JAMA Network Open*

eReferences

1. Zima BT, Gay JC, Rodean J, et al. Classification System for International Classification of Diseases, Ninth Revision, Clinical Modification and Tenth Revision Pediatric Mental Health Disorders. *JAMA Pediatr*. 2020;174(6):620. doi:10.1001/jamapediatrics.2020.0037
2. Mental Health Disorder Codes. Published 2023. https://www.childrenshospitals.org/content/analytics/toolkit/mental-health-disorder-codes
3. Healthcare Cost and Utilization Project. CENTRAL DISTRIBUTOR SEDD: DESCRIPTION OF DATA ELEMENTS. https://hcup-us.ahrq.gov/db/vars/sedddistnote.jsp?var=hispanic
4. Duncan AF, Montoya-Williams D. Recommendations for Reporting Research About Racial Disparities in Medical and Scientific Journals. *JAMA Pediatr*. Published online January 2, 2024. doi:10.1001/jamapediatrics.2023.5718
5. Urban Influence Codes. Published 2023. Accessed November 8, 2023. https://www.ers.usda.gov/data-products/urban-influence-codes/
6. Hedegaard H, Johnson RL, Garnett M, Thomas KE. The International classification of diseases, 10th revision, clinical modification (ICD–10–CM): external cause-of-injury framework for categorizing mechanism and intent of injury. *Natl Health Stat Report*. Published online 2019.
7. Clinical Classifications Software Refined (CCSR). Healthcare Cost and Utilization Project (HCUP). Published 2022. Accessed November 8, 2023. www.hcup-us.ahrq.gov/toolssoftware/ccsr/ccs_refined.jsp

© 2024 Kemal S et al. *JAMA Network Open*
