# Supplementary material for: Emergency Department Use by Youths Before and After Self-Inflicted Intentional Injury
Source: JAMA Netw Open. 2024 Aug 15;7(8):e2427350. doi: 10.1001/jamanetworkopen.2024.27350 (PMC11327883; doi:10.1001/jamanetworkopen.2024.27350)
Supplement: Supplement 2. — Data Sharing Statement [file jamanetwopen-e2427350-s002.pdf]

Data Sharing Statement

Kemal. Emergency Department Use by Youths Before and After Self-Inflicted Intentional Injury. *JAMA Netw Open*. Published August 15, 2024. doi:10.1001/jamanetworkopen.2024.27350

Data
Data available: No

Additional Information
Explanation for why data not available: Our data was obtained from the Agency for Healthcare Quality and Research Healthcare Cost and Utilization Project whose data is available for purchase through their platform.
